# Comparative Genomics Reveals 13 Different Isoforms of Mytimycins (A-M) in *Mytilus galloprovincialis*

**DOI:** 10.3390/ijms22063235

**Published:** 2021-03-22

**Authors:** Magalí Rey-Campos, Beatriz Novoa, Alberto Pallavicini, Marco Gerdol, Antonio Figueras

**Affiliations:** 1 Institute of Marine Research (IIM), CSIC, Eduardo Cabello 6, 36208 Vigo, Spain; mrey@iim.csic.es (M.R.-C.); beatriznovoa@iim.csic.es (B.N.); 2 Department of Life Sciences, University of Trieste, Via Giorgieri 5, 34127 Trieste, Italy; pallavic@units.it; 3 National Institute of Oceanography and Applied Geophysics–OGS, via Auguste Piccard, 54, 34151 Trieste, Italy

**Keywords:** *Mytilus galloprovincialis*, mytimycins, mussel genome, RNA-seq, isoelectric point, positive and negative selection, promoter

## Abstract

Mytimycins are cysteine-rich antimicrobial peptides that show antifungal properties. These peptides are part of the immune network that constitutes the defense system of the Mediterranean mussel (*Mytilus galloprovincialis*). The immune system of mussels has been increasingly studied in the last decade due to its great efficiency, since these molluscs, particularly resistant to adverse conditions and pathogens, are present all over the world, being considered as an invasive species. The recent sequencing of the mussel genome has greatly simplified the genetic study of some of its immune genes. In the present work, we describe a total of 106 different mytimycin variants in 16 individual mussel genomes. The 13 highly supported mytimycin clusters (A–M) identified with phylogenetic inference were found to be subject to the presence/absence variation, a widespread phenomenon in mussels. We also identified a block of conserved residues evolving under purifying selection, which may indicate the “functional core” of the mature peptide, and a conserved set of 10 invariable plus 6 accessory cysteines which constitute a plastic disulfide array. Finally, we extended the taxonomic range of distribution of mytimycins among Mytilida, identifying novel sequences in *M. coruscus*, *M. californianus*, *P. viridis*, *L. fortunei*, *M. philippinarum*, *M. modiolus*, and *P. purpuratus*.

## 1. Introduction

Mytimycins are cysteine-rich antimicrobial peptides isolated for the first time from *Mytilus edulis* in 1996 [1]. Like several other antimicrobial peptides (AMPs), mytimycins contain several conserved cysteines in the mature peptide, whose connectivity is still unclear [1,2]. Since these molecules show antifungal properties [3], they have been notably less studied than other mussel antibacterial and antiviral peptides. Although multiple mytimycin variants have been previously reported in *Mytilus galloprovincialis* so far [4], only a single variant, described in 2011, has been the subject of detailed studies [4]. This AMP is produced as a precursor peptide, which includes a signal peptide of 23 amino acids, a mature peptide of 54 amino acids (containing 12 cysteines), and a C-terminal extension of 75 amino acids, which contains an EF hand-motif [4].

The mytimycin gene is mainly expressed in circulating hemocytes, the central immune cells of mussels. These AMPs are strongly up-regulated after a stimulation with the fungus *Fusarium oxysporum* [5]. However, the relevant inter-individual differences in response to specific stimuli in these animals [6] have hampered the definition of consistent patterns of regulation for the mytimycin gene [7]. Mytimycins are part of the complex immune network that constitutes the defense system of the Mediterranean mussel, along with other AMPs such as defensins, myticins, or mytilins [8,9,10]. These, in association with other recognition and effector molecules, create a highly efficient immune system, which likely prevents the massive mortality events that often occur in the natural environment for other bivalve species [11,12]. The advance of massive sequencing technologies has provided a strong contribution in unveiling this scenario, in particular thanks to the release of the *Mytilus galloprovincialis* genome sequence [13], which revealed the presence of widespread gene presence/absence variation for the first time in a metazoan. This dispensable nature of 25% of the mussel coding genes undoubtedly offers a source of enormous genetic variability. A striking example of this molecular diversity is represented by another class of cysteine-rich AMPs, i.e., myticins, which show a great level of inter-individual variability, with very little overlap among individuals (120 different isoforms were described in only 16 individuals) [14].

The main objective of this work was to continue studying the molecular diversity of the immune genes of mussel *Mytilus galloprovincialis*, extending investigations to the case of mytimycins. We explored the sequence variants belonging to this gene family, exploiting the availability of a reference genome and resequencing data of 16 different individuals [13]. We also investigated several previously unexplored aspects that might help to further characterize the biological roles of these molecules, such as their isoelectric point, the regulatory elements present in the promoter region of the gene, and their expression in different animals and tissues.

## 2. Results

### 2.1. Searching, Screening, and Identifying M. galloprovincialis Mytimycins

A total of 106 different nucleotide sequences encoding mytimycins were found in the 16 mussel genome assemblies, i.e., all the mytimycin variants that were present in the analyzed genomes. These 106 sequences code 94 different peptides, 76 of which are mytimycins with an uninterrupted CDS and 18 of which are pseudogenes (sequences that incorporate a STOP codon which interrupts the open reading frame) (File S1).

### 2.2. Phylogenetic Analysis

**Figure 1:**
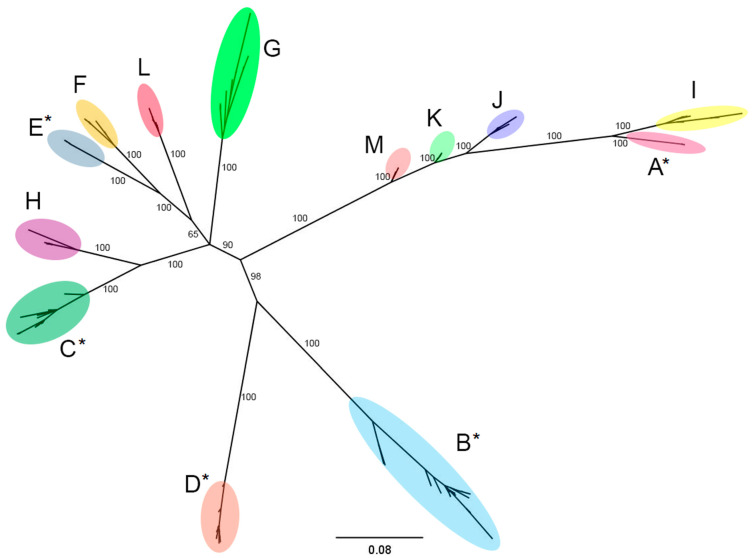
Phylogenetic analysis. The full coding region of 106 mytimycin sequences obtained from 16 genomes were subjected to multiple sequence alignment and analyzed with Bayesian phylogenetic inference. The employed evolution model was the Hasegawa, Kishino, and Yano 1985 model (HKY + G). Thirteen clusters of sequences are defined with high posterior probability support. * indicates previously described isoforms.

The Bayesian phylogenetic tree of all the 106 mytimycin variants identified in this study (Figure 1) displayed a remarkable subdivision of mytimycins in 13 groups (A–M), with well-supported branches posterior probabilities. All the sequences grouped within the same cluster showed a pairwise identity threshold higher than 95% (File S1) and displayed a variable number of cysteine residues, as will be reported in detail below along with the report of mytimycins in other Mytilida. An alignment of the peptide sequence of all the variants is shown in the Figure S1.

### 2.3. Presence/Absence Variation

An evaluation of the presence/absence of all the 106 sequences was performed in the 16 mussel genomes (File S2). On average, each mussel genome showed nine different mytimycin sequences, with each of the 16 individuals analyzed displaying an average of five unique variants (i.e., variants not present in any of the other 15 genomes). This fact highlights a scenario of enormous diversity, which results in a unique collection of mytimycin variants in each mussel. The sequences which displayed the highest frequency of occurrence are F1, F2, E1, and J1. Even so, none of them were present in all the genomes analyzed.

**Figure 2:**
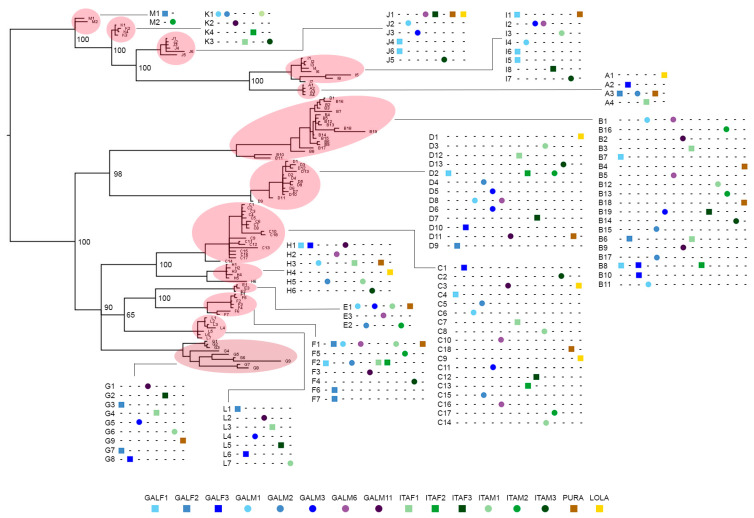
Presence/absence variation cladogram. The graph shows the presence/absence variation of each mytimycin nucleotide variant in each mussel genome, along with the arbitrarily rooted phylogenetic tree shown in Figure 1. Numbers close to each node are posterior probabilities.

As several of the 106 variants identified only displayed minor differences in pairwise comparisons, we cannot exclude the possibility that they represent polymorphic alleles of the same gene. To investigate this possibility, we generated a cladogram showing which variants were present in each genome (Figure 2).

**Figure 3:**
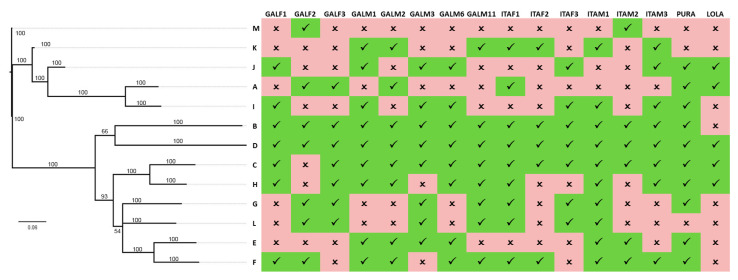
Clusters presence/absence variation. Matrix shows presence/absence of the 13 mytimycin clusters in all genomes, along with a Bayesian phylogenetic tree. Numbers close to each node are posterior probabilities.

To verify to which extent mytimycins were subjected to presence/absence variation, we used a maximum parsimony approach, tentatively assigning all the 106 sequence variants to the 13 clusters identified by the phylogenetic analysis, under the assumption that each cluster may include different allelic variants belonging to the same gene. This approach confirmed that PAV was the most likely explanation for the observed patterns of distribution, as different mytimycin clusters displayed largely different frequencies of occurrence (Figure 3). Notably, the clusters belonging to a monophyletic branch of the tree (MKJAI) were generally absent in genomes, whereas cluster D was present in all genomes, and B and C were found in 15 out of the 16 studied genomes.

### 2.4. Isoelectric Point

**Figure 4:**
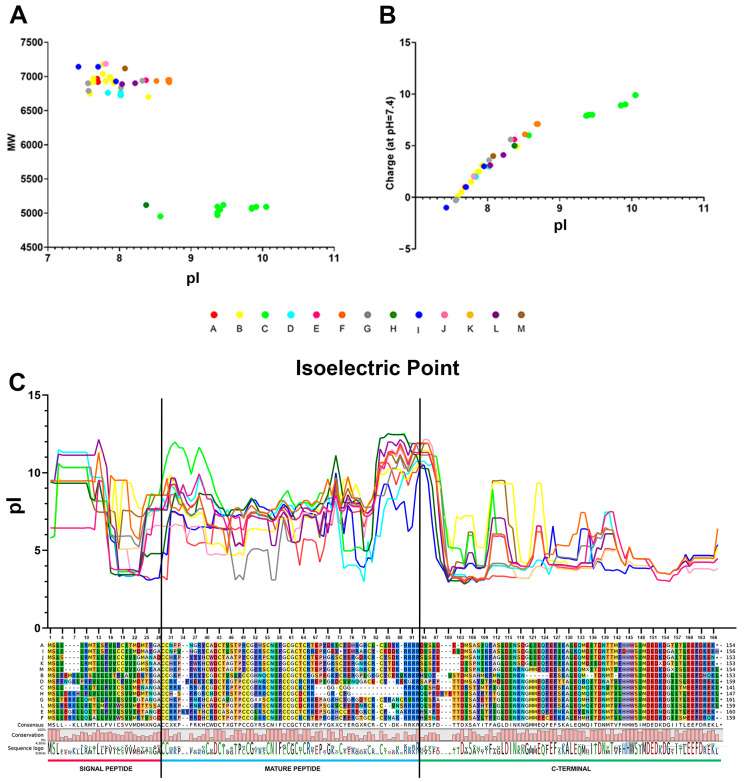
Isoelectric Point. (**A**) Isoelectric point (X axis) and molecular weight (Y axis) of the mature peptide of mytimycins with an uninterrupted CDS. (**B**) Isoelectric point (X axis) and charge at pH = 7.4 (Y axis) of the mature peptide of mytimycins with an uninterrupted CDS. (**C**) Isoelectric point of the whole sequence of the 13 mytimycin isoforms (consensus sequence of each isoform). The isoelectric point distribution was analyzed through the calculation of the average isoelectric point based on a sliding window of 15 amino acids. The alignment shows the consensus sequence of the 13 mytimycins clusters obtained from 16 genomes. X represents polymorphic amino acid residues found in each cluster. * represents STOP codons.

The isoelectric point and predicted charge at cytoplasmic pH (i.e., 7.4) of all the mytimycins with an uninterrupted CDS were obtained (mature peptide), and they are reported in File S3. Figure 4A shows that the mature peptides of almost all the mytimycins display very narrow variations in terms of isoelectric point (pI), which varies between 7 and 9. However, the C isoform has a slightly higher pI (between 9 and 10). In terms of charge, the mature peptides are usually cationic, with a positive net charge varying from 0 to 10 (Figure 4B), with the C isoform once again showing the highest values.

The sliding-window analysis of pI along the whole peptide, carried out on the 13 representative precursor peptides of mytimycins (Figure 4C), showed very similar profiles, with a notable decrease at the C-terminal region, characterized by the presence of several acidic residues.

### 2.5. Positive and Negative Selection Analysis

**Figure 5:**
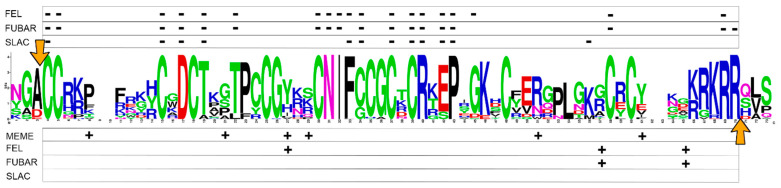
Positive and negative selection analysis. The mature peptide of the mytimycins with an uninterrupted CDS were selected to perform the analysis. Four prediction models have been used (MEME, FEL, FUBAR, and SLAC). Positive selection sites and negative selection sites are collected on the graph, using +/− symbols respectively. Orange arrows point to the beginning and end site of the mature peptide, marking the signal peptide and propeptide cleavage sites, respectively.

The selection analysis mostly identified sites subject to negative selection in the mature peptide region. Despite some minor differences, the various tests were concordant in recognizing a major block of residues under pervasive purifying selection between positions 30 and 43 in the multiple sequence alignment (Figure 5). These sites match with four of the cysteines that form the characteristic disulfide array of mytimycins and could constitute the “core functional region” of these peptides. Some other cases of negatively selected codons were also identified along the mature peptide regions, mostly corresponding to conserved cysteines. Moreover, the 3′ end of the mature peptide region displays signatures of negative selection in conjunction with a dibasic site, which may be recognized as a furin-like proteolytic cleavage site of the precursor protein. On the other hand, the few (i.e., 8 out of 67) codons under positive selection were spread along the mature peptide region and they were often accompanied by low statistical support (*p*-value < 0.09).

### 2.6. Promoter Analysis

**Figure 6:**
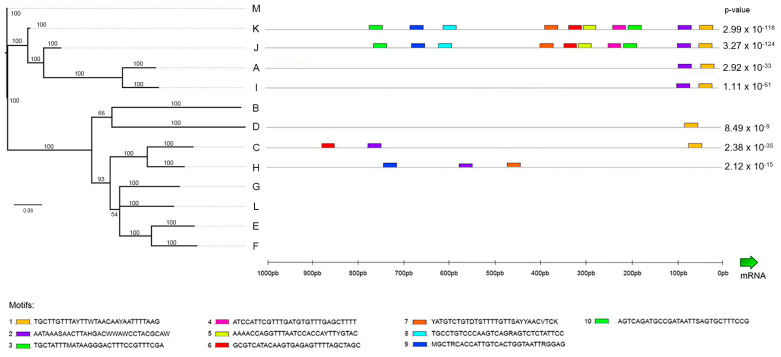
Promoter analysis. The promoter (1 kb) of 7 complete available isoforms was analyzed. Colored boxes show the 10 different significant motifs found. The p-value of the combined position of all motifs present in each sequence is also shown.

The promoter analysis identified 10 different motifs located in the 1000 pb upstream of the gene transcription start site (Figure 6). The most conserved motif shared by almost all the available sequences among the 13 clusters (i.e., the promoter regions could not be retrieved in some cases due to the excessive fragmentation of the genome assemblies) was the motif 1 (TGCTTGTTTAYTTWTAACAAYAATTTTAAG). This motif was only missing in the H cluster, which included several sequences with in-frame stop codons, and therefore is likely pseudogenic (Figure S1). The two phylogenetically close clusters J and K (Figure 1) shared several other motifs that could not be found in any of the other clusters. In general, mytimycin genes do not appear to show a robust promoter, as evidenced by the lack of conserved motifs in fixed relative positions.

### 2.7. Genomic vs. Transcriptomic Data

Having the genome and transcriptome of the same individual offers a great opportunity to investigate RNA editing processes which may occur after the transcription [13]. Six different mytimycin sequence variants were found in the reference genome (LOLA) (File S2) and only four of these were present in the transcriptome assembly, indicating that two of the genomic forms were not expressed. The comparison between the genomic sequences and mRNA sequences of the four expressed mytimycin variants highlighted that there were no discrepancies, allowing us to disregard mRNA editing as a plausible phenomenon responsible for the molecular diversity observed in this family of AMPs.

### 2.8. Expression Analysis

**Figure 7:**
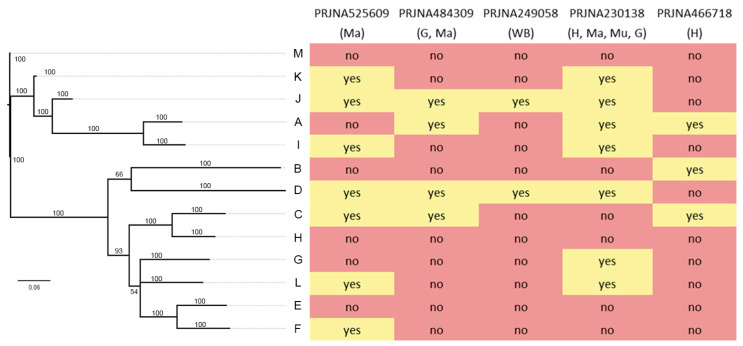
Expression analysis. A total of five transcriptomes of different tissues and mussel locations were analyzed to find evidence of expression of sequences belonging to each cluster. The isoforms expressed in each transcriptome are highlighted in yellow. Red color indicates no evidence of expression.

Five different transcriptomes from different mussel tissues and geographical locations were analyzed to investigate whether sequences belonging to the 13 previously defined clusters were broadly expressed (Figure 7). Some of these variants, such as those from clusters D and J, were broadly expressed across the different samples analyzed (e.g., in 4 out of the 5 transcriptomes analyzed). Others were scarcely expressed or, as in the case of the M, H, and E clusters, not expressed at all in any of the studied transcriptomes.

### 2.9. Taxonomical Distribution of Mytimycins

Although the -omic information available for marine mussels is still incomplete, the screening of available genomes and transcriptomes allowed us to extend their range of distribution beyond *M. galloprovincialis* and *M. edulis* [1,3]. We showed that mytimycins are found in *M. coruscus* and *M. californianus*, belonging to the same genus, but not inter-fertile with the other species included in the *M. edulis* species complex. Mytimycins were also present in *Perna viridis* (subfamily Mytilinae) [15], in *Limnoperna fortunei* (subfamily Arcuatulinae) [16], in *Modiolus philippinarum* and *Modiolus modiolus* (subfamily Modiolinae) [17], and in *Perumytilus purpuratus* (subfamily Brachidontinae) [18], but not in Bathymodiolinae [17].

The mytimycin sequences detected in the different mytilid subfamilies widely differed due to the number of cysteine residues, which varied from 10 to 14, and due to the type of disulfide array present (Table 1). Mytimycins adopted five possible types of disulfide arrays, with only three of them (i.e., type I, type II, and type III) found in *M. galloprovincialis*. Type II array, found in the A, I, J, and K groups within *M. galloprovincialis*, denoted the more widespread architecture, found in all Mytilida, except *L. fortunei*. Curiously, the type I array, characterizing groups B, D, E, F, G, L, and M in the Mediterranean mussel, was exclusively present in *Mytilus* spp. Type III array was only shared by *Mytilus* spp. and *L. fortunei*, whereas the type IV array, found in three different mytilid subfamilies, was not present in *M. galloprovincialis* and its congeneric species. Finally, a particular disulfide array with 14 cysteine residues was only found in sequences of the golden mussel.

**Figure 8:**
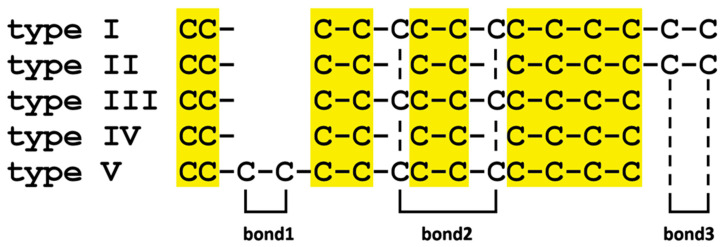
Predicted disulfide connectivity based on in silico analyses. The 10 cysteine residues which compose the backbone of mytimycins are highlighted with a yellow background.

Based on the multiple alignment of the sequences, and on the presence/absence of paired cysteine residues in the five aforementioned types of disulfide arrays, it was possible to tentatively predict the connectivity of a few cysteine residues, even though the three-dimensional structure of mytimycins currently remains unsolved. These considerations are limited to the three optional disulfide bonds, whereas the connectivity among the 10 cysteine residues which characterize the backbone of mytimycins are yet to be investigated (Figure 8).

The three optional bonds are as follows: (i) bond 1 connects two consecutive cysteine residues found towards the N-terminal portion of the mature peptide of type V mytimycins, between the 2nd and 3rd Cys of the backbone array; (ii) bond 2 is found in type I, III, and V mytimycins; this disulfide bond connects the two optional cysteine residues found in tandem with the 5th and 7th Cys residues of the backbone array, and (iii) bond 3 is only found in type I and type II mytimycins and connects the two cysteine residues found at the C-terminal end of the mature peptide.

**Figure 9:**
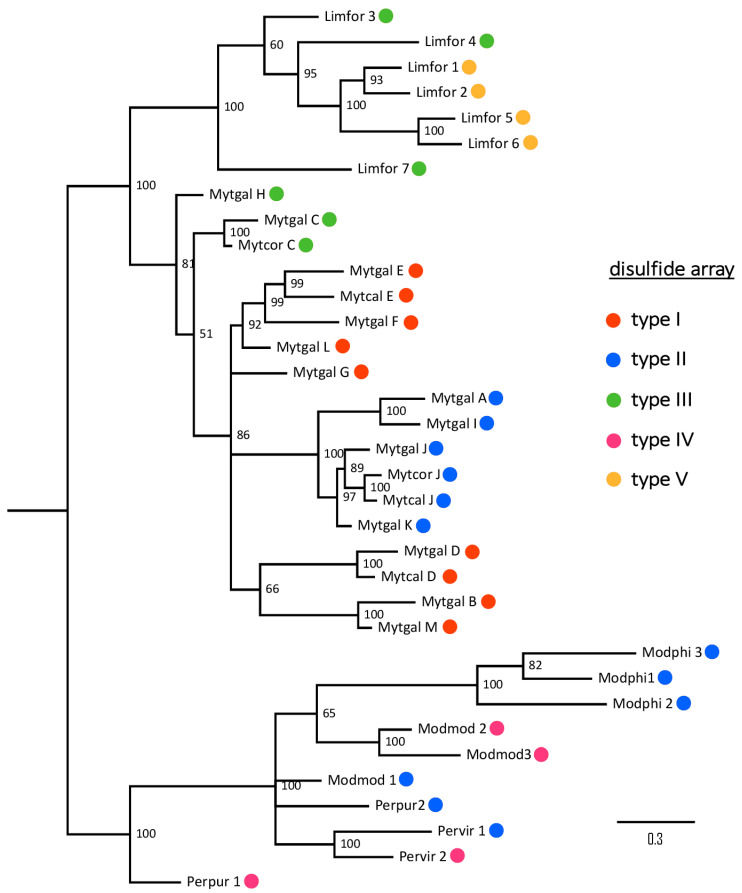
Bayesian phylogeny (run under a WAG+G model of molecular evolution, with a MCMC analysis with 500,000 generations) of the mytimycin sequences identified in different Mytilida species. Posterior probability support values are shown next to each node. Colored dots indicate the type of disulfide array observed in each sequence.

From an evolutionary perspective, the phylogenetic clustering of mytimycins was based on taxonomy rather than on the type of disulfide array (Figure 9). The observed topology of the tree strongly hints that different disulfide arrays have been independently acquired in the three main clades.

## 3. Discussion

In the past few years, the genomic and transcriptomic study of *Mytilus galloprovincialis* has revealed an enormous amount of inter-individual genetic diversity in this bivalve of great economic and ecological interest [6,13]. The most curious feature recently unmasked by the study of its genome is the phenomenon of gene presence/absence variation, described for the first time in a metazoan, as it implies that each individual is endowed with a particular and exclusive repertoire of genes, different from all the other individuals of the same species [13]. Several AMPs are included among the high number (i.e., >20,000) of dispensable genes encoded by the mussel genome, making them very interesting subjects of study due to their possible involvement in resistance to pathogenic infections [11,12]. Although the molecular diversity of other AMPs such as mytilins [19] and myticins [14] has been recently investigated, this aspect had not been investigated yet for mytimycins.

Our screening of *M. galloprovincialis* genomic data identified 106 unique variants, which could be clustered based on pairwise similarity and phylogeny in 13 well-supported clusters. Just five of these (cluster A, [1]; clusters B, C, D, and E, [4]) had been previously described and the other eight (mytimycin F–M) are here reported as entirely novel variants. The comparative analysis of 16 individual genomes confirmed that PAV plays a major role in shaping the exceptional level of inter-individual sequence diversity in this AMP family (Figure 3), whereas RNA editing processes are likely not involved.

Some of the isoforms described in this study (i.e., 18 out of 106) incorporated in-frame STOP codons, which is in line with the tendency of some recently duplicated AMP gene copies to undergo pseudogenization [20]. Cluster H, which mostly comprises sequences including STOP codons, with poor conservation of regulatory elements in the promoter region (Figure 6) and lack of expression (Figure 7), is a major candidate as a non-functional evolutionary relict. In this sense, a poorly conserved promoter in the mytimycin array, coupled with the large variability described, might suggest a scenario of balancing selection by which multiple alleles are maintained in the population, and, at the same time, there is strong selection in favor of a small number of shared characteristics.

We showed that the different mytimycin variants identified in *M. galloprovincialis* and other Mytilid species were all characterized by the presence of a core set of 10 conserved cysteine residues, invariably present in the mature peptide. Six accessory cysteine residues, only found in some variants, may provide up to three extra intra chain disulfide bonds, resulting in the five alternative disulfide arrays reported in Figure 8. In absence of structural data, it is presently unknown whether the typical cysteine stabilized alpha-beta (CSαβ) folding found in other mussel AMPs, such as defensins, mytilins, and myticins, is also present in mytimycins. In any case, the presence of multiple disulfide connectivity suggests that mytimycins may be subject to a certain degree of structural plasticity, like other mussel AMPs including mytimacins [21], pseudomyticins [14], and mytilins [19].

From an evolutionary perspective, the phylogenetic clustering of mytimycins based on taxonomy rather than on the type of disulfide array was quite surprising (Figure 9). The observed topology of the tree strongly hints that different disulfide arrays have been independently acquired in the three clades through a process of convergent evolution, starting from an unknown ancestral cysteine framework, which likely included the 10 hyper-conserved residues found in all mytimycins. Hence, the gain of the accessory disulfide bond 3 in the type II array (Figure 8) is expected to have occurred by independent means in *Mytilus* spp. and in the *Modiolus/Perna/Perumytilus* clade (Figure 9). On the other hand, other events, such as the acquisition of the accessory disulfide bond I in the type V array, as well as the acquisition of the accessory disulfide bond 2 in the type I array, mark lineage-specific features of *L. fortunei* and *Mytilus* spp., respectively. The lack of significant similarities between the mature region of mytimycin and other cysteine arrays present in the genomes and transcriptomes of other bivalves, together with the lack of mytimycin genes in Bathymodiolinae, suggest that these AMPs are taxonomically restricted to the order Mytilida. The future availability of -omic resources for other key missing subfamilies (e.g., Crenellinae and Musculinae) might help to further clarify these aspects.

The conserved disulfide array, along with the unknown 3D folding it provides, may be the key to explaining the peculiar antifungal properties of mytimycins [3]. Interestingly, some cysteine residues were buried within a highly conserved block of amino acids located in the central part of the mature peptide, which displays strong evidence of negative selection. We believe that this “central core” represents an interesting target for future functional studies.

Like other mussel AMPs, the precursors peptides of mytimycins display an anionic C-terminal extension, which immediately follows a dibasic site that might serve as a proteolytic cleavage site (Figure 4C) [14,19]. However, unlike other mussel AMPs, this region is not devoid of a defined 3D structure, but contains an EF-hand domain. This structural module is characterized by a helix-loop-helix motif and it is involved in binding intracellular calcium [22,23]. In eukaryotic cells, this domain is usually found in proteins involved in calcium signaling [23]. While Ca^2+^ is crucial in the cardiac excitation-contraction coupling [24,25], it also participates in many other important biological processes, including immune functions. Proteins containing an EF-hand domain are thought to be an important regulator of T cell cytotoxicity [26]; this domain is also found in calmodulin-like 6 (CALML6), where it suppresses antiviral response by preventing the translocation of *IRF3* (interferon regulatory factor (3)) into the nucleus and inhibiting the IFN pathway [27]. While the functional meaning of the EF-hand domain in the mytimycin precursor peptides remains unknown, it is tempting to speculate that it could be involved in innate immune response to pathogens, activating pattern recognition receptors and mediating inflammation processes [28]. The mytimycin EF-hand domain, highly conserved between variants and across species, has been likely acquired along evolution from a pre-existing gene encoding an EF-hand domain-protein (highly abundant in mytilid genomes) [13]. Curiously, a single sequence among those identified in this study entirely lacks this region, along with the dibasic cleavage site that marks the boundary between the mature peptide and the pro-peptide region. Whether this sequence (identified in *P. purpuratus*) represents an ancestral prototypical mytimycin (i.e., before the acquisition of the EF-hand domain encoding exon), or rather the secondary product of an exon loss event, remains to be investigated.

In summary, this work provides a complete overview of the molecular diversity of mytimycins both within species (using *M. galloprovincialis* as a reference) and between different species of Mytilida, extending the taxonomical range of distribution of these AMPs and revealing the important role of PAV in determining the individual repertoire of antifungal peptides encoded by the genomes of each individual. Other important biological aspects remain to be clarified, including the functional relevance of the highly conserved and negatively selected core of the mature peptide, and the mode of regulation of the expression of mytimycin genes, which could not be elucidated due to the scarce number of conserved elements present in the promoter regions.

## 4. Materials and Methods

### 4.1. Searching, Screening, and Identifying M. galloprovincialis Mytimycins

The recently published mussel genome [13] served as database to screen the whole set of mytimycins variants present in the 16 individuals sequenced within the frame of the aforementioned project.

The 16 available genomes were labeled as follows: LOLA (the reference genome), PURA, GALF1, GALF2, GALF3, GALM1, GALM2, GALM3, GALM6, GALM11 (from Galicia), ITAF1, ITAF2, ITAF3, ITAM1, ITAM2, and ITAM3 (from Italy), being “F” and “M”, female and male mussels, respectively. LOLA and PURA are also female individuals.

The mytimycin sequence previously described by Sonthi et al. in 2011 [3] was used as seed to perform tBLASTn searches against BLAST databases of all the previously listed genomes with the CLC Genomics Workbench v20.0.3 (Qiagen, Hilden, Germany). The e-value threshold was set at 1e-5 and resulting hits were manually checked to remove false positives.

MEGA-X Software environment v10.1.7 (https://www.megasoftware.net/, accessed on 1 February 2021) [29] served as a tool to align the sequences using the MUSCLE method [30].

All the sequences were arbitrarily clustered in 13 different groups (A–M) according to their sequence identity, and a consensus sequence was then established for each cluster, enabling further downstream analyses.

### 4.2. Phylogenetic Analysis

The whole set of nucleotide sequences was taken into account to find the best suitable model of molecular evolution. jModelTest v3.7 (http://evomics.org/learning/phylogenetics/jmodeltest/, accessed on 1 February 2021) [31,32] was the software used for this purpose, identifying the Hasegawa, Kishino, and Yano 1985 model [33], with a Gamma-distributed rate of variation across sites (HKY+G), as the best-fitting model based on the corrected Akaike information criterion.

A Bayesian inference analysis was run with a Monte Carlo Markov Chain approach using the MrBayes v3.2.7 software (http://nbisweden.github.io/MrBayes/, accessed on 1 February 2021) [34]. Two independent analyses with four chains each were run in parallel for 800,000 generations until the effective sample size estimated for all the parameters of the model reached a value >200. The resulting phylogenetic tree was graphically represented using FigTree v1.4.4 (http://tree.bio.ed.ac.uk/software/figtree/, accessed on 1 February 2021) [35]. By the same approach, a simplified phylogenetic analysis was also performed using the established consensus sequences for each of the 13 clusters of mussel mytimycins.

### 4.3. Isoelectrical Point

SignalP v3.0 (http://www.cbs.dtu.dk/services/SignalP-3.0/, accessed on 1 February 2021) [36] was the software used to detect the signal peptide cleavage site and to predict the N-terminal end of the mature peptide. Due to the unknown nature of the protease involved in the cleavage of the C-terminal region of mytimycin precursors, the putative C-terminal end of the mature peptide was identified based on the alignment with the known mature peptides previously described by other authors [3].

The isoelectric point (pI) and the charge of the mature peptide (at cytoplasmic pH = 7.4) of all the mytimycins were calculated using the Isoelectric Point Calculator software (http://isoelectric.org/, accessed on 1 February 2021) [37]. Moreover, the charge distribution of the complete peptide (consensus sequences) of the 13 groups of mytimycins (A–M) was analyzed through the calculation of the average pI based on a sliding window of 15 amino acids.

### 4.4. Positive and Negative Selection Analysis

Datamonkey Adaptive Evolution Server (https://www.datamonkey.org/, accessed on 1 February 2021) [38] was the software used to detect sites evolving under positive and negative selection. Specifically, the codon-aligned nucleotide sequences of the mature peptide coding region of all the mytimycins with an uninterrupted CDS were analyzed to detect sites evolving under episodic positive selection with the MEME algorithm [39], as well as pervasive positive/negative selection using the FEL [40], FUBAR [41], and SLAC [40] algorithms.

### 4.5. Promoter Analysis

The previously identified coding region matches were used as a seed to extend the reconstruction of full mytimycin genes from the genome assemblies, retrieving the sequences of the exon 1 (no coding 5′-UTR region) whenever possible. The Genie tool (https://www.fruitfly.org/index.html, accessed on 1 February 2021) [42] was used to predict the 5′ splicing acceptor sites and the 3′ splicing donor sites and thereby to define the boundaries of exons. Once the full gene structure was appropriately annotated, we extracted 1000 bp upstream of the 5′ end of the first exon to perform a promoter analysis by searching for conserved ungapped motifs shared by most mytimycin genes. The software used for this purpose was MEME Suite v5.1.1 (https://meme-suite.org/meme/doc/download.html, accessed on 1 February 2021) [43], setting the accepted length of such motifs between 6 and 30 bp.

### 4.6. Genomic vs. Transcriptomic Data

The cited Mussel Genome Project [13] included the transcriptome sequencing data from LOLA (the reference individual). Therefore, genome and transcriptome of the same mussel were available, allowing the comparison between genomic DNA and mRNA sequences. Using the same methodology described above for genomic sequences, mytimycin-coding transcripts were retrieved from the transcriptome assembly and used as a reference for mapping the Illumina reads deriving from genome sequences. This approach allowed us to detect the presence of differences between DNA and RNA of the same individual. The mapping parameters were set to be highly stringent in order to only allow perfect matches (length fraction = 1 and similarity fraction = 1). The mapping files obtained were then visually inspected to detect regions with no read coverage, which could indicate mismatches between the genomic DNA and mRNA sequences and pinpoint the presence of sites subjected to RNA editing.

### 4.7. Expression Analysis

The SRA-NCBI (Sequence Read Archive-National Center for Biotechnology Information) database was inspected in order to find the available mussel transcriptomic datasets, and five different transcriptome assemblies of *M. galloprovincialis* from different geographic locations and different tissues were found. In detail, the transcriptomes included in the present study were PRJNA525609 (mantle), PRJNA249058 (whole-body), PRJNA484309 (gill and mantle), PRJNA230138 (hemocytes, mantle, muscle, and gill), and PRJNA466718 (hemocytes). These resources were used to find evidence of mytimycin expression, screening the de novo assembled transcriptomes (generated with the CLC Genomics Workbench) with a tBLASTn approach and using the coding region of the 13 aforementioned mytimycin clusters as queries, as previously described. Matches that shared >95% identity with the query sequence were considered as evidence of the expression of a mytimycin variant belonging to the underlying sequence cluster.

### 4.8. Taxonomical Distribution of Mytimycins

In order to study the taxonomic range of distribution of mytimycins in Mytilida mussels, mytimycin sequences were retrieved using the same tBLASTn-based approach described above in the transcriptomes of two other species belonging to the *Mytilus* genus, i.e., *M. coruscus* (genome assembly PRJEB33342) and *M. californianus* (transcriptome assembly PRJNA37512). This strategy was extended to other Mytilida, allowing the identification of mytimycins in *Perna viridis* (subfamily Mytilinae) [15], in *Limnoperna fortunei* (subfamily Arcuatulinae) [16], in *Modiolus philippinarum* and *Modiolus modiolus* (subfamily Modiolinae) [17], and in *Perumytilus purpuratus* (subfamily Brachidontinae) [18]. These identified sequences are reported in the File S4. To investigate the evolutionary relationships between the mytimycins of different mussel species, a Bayesian phylogenetic inference analysis was performed under a WAG+G model of molecular evolution [44], with a MCMC analysis with 500,000 generations.

## Figures and Tables

**Table 1:** Taxonomic distribution of the mytimycin cysteine arrays observed in Mytilida. For *M. galloprovincialis*, the type

Cys Array	Details	Cys Residues	*M. galloprovincialis*	*Mytilus* spp	*P. viridis*	*Modiolus* spp	*L. fortunei*	*P. purpuratus*	*B. platifrons*
type I	CC- C-C-CC-C-CC-C-C-C-C-C	14	√ (B, D, E, F, G, L, M)	√	×	×	×	×	×
type II	CC- C-C- C-C- C-C-C-C-C-C	12	√ (A, I, J, K)	√	√	√	×	√	×
type III	CC- C-C-CC-C-CC-C-C-C	12	√ (C, H)	√	×	×	√	×	×
type IV	CC- C-C- C-C- C-C-C-C	10	×	×	√	√	×	√	×
type V	CC-C-C-C-C-CC-C-CC-C-C-C	14	×	×	×	×	√	×	×

## Data Availability

Not applicable.

## Supplementary Materials

The following are available online at https://www.mdpi.com/1422-0067/22/6/3235/s1, File S1: Mytimycin database; File S2: Presence/absence evaluation. The matrix shows the presence and absence of each mytimycin sequence (106 different sequences) in each mussel genome (16 different genomes); File S3: Isoelectric point analysis. The mature peptide of the whole set of mytimycins with an uninterrupted CDS was analyzed. Values of molecular weight, isoelectric point, and charge are available; File S4: Mytilida sequences; Figure S1: Alignment of the peptide sequence of all the mytimycin variants.
