# Supplementary figures and images for: Comparative Genomics Reveals 13 Different Isoforms of Mytimycins (A-M) in Mytilus galloprovincialis
Source: Int J Mol Sci. 2021 Mar 22;22(6):3235. doi: 10.3390/ijms22063235 (PMC8004829; doi:10.3390/ijms22063235)

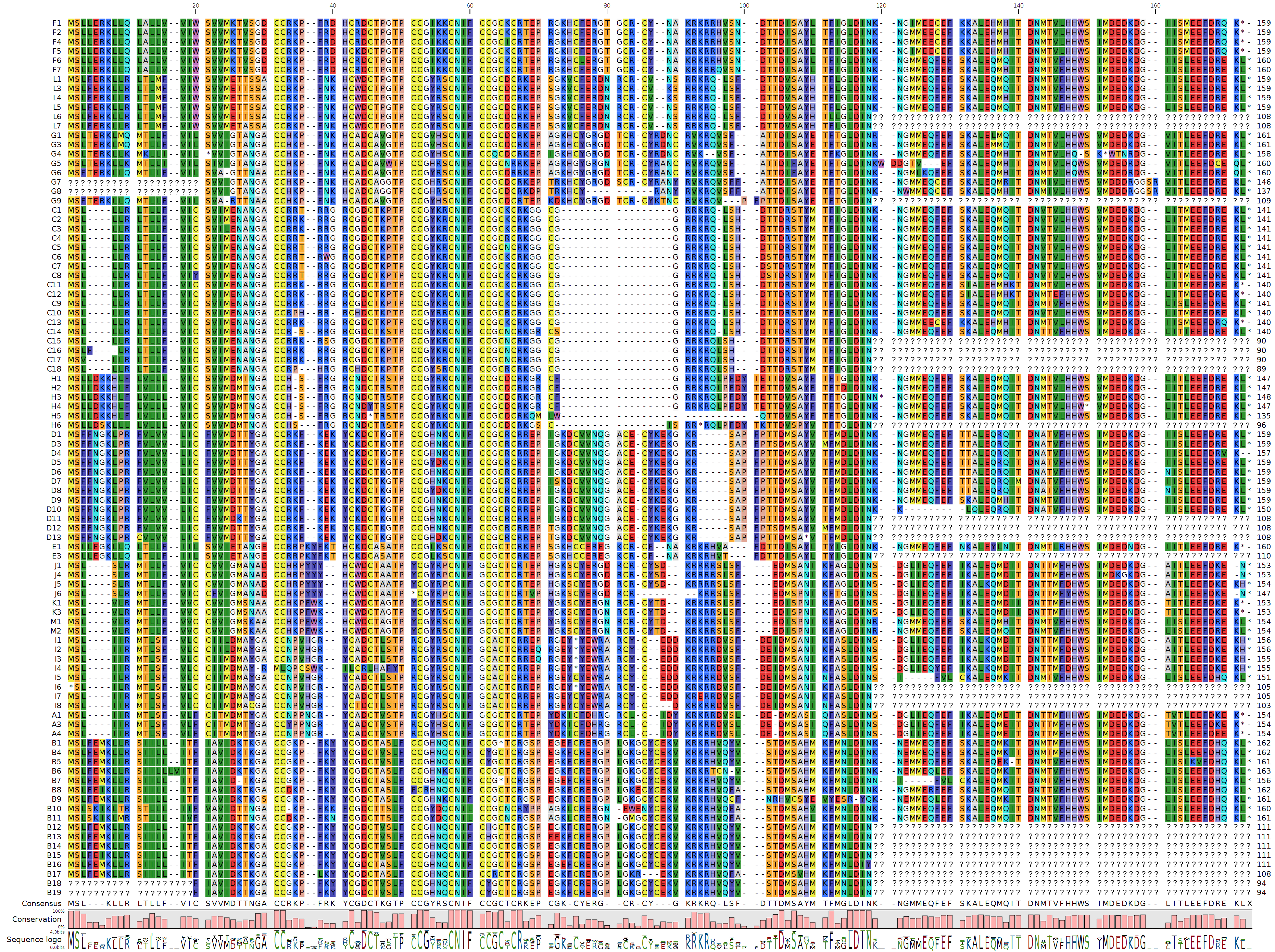

Supplement: Supplementary file 1 [file ijms-22-03235-s001.zip › ijms-1114682-submit-supp/Figure S1.tif]
